# The effects of different feeding strategies providing different levels of vitamin A on animal performance, carcass traits, and the conversion rate of subcutaneous fat color in cull-cows

**DOI:** 10.1093/tas/txae071

**Published:** 2024-04-27

**Authors:** J T Parkinson, H J Cochran, J D Kieffer, A E Relling, S L Boyles, R E Kopec, L G Garcia

**Affiliations:** Department of Animal Sciences, The Ohio State University, Columbus, OH 43210, USA; Department of Animal Sciences, The Ohio State University, Columbus, OH 43210, USA; Department of Animal Sciences, The Ohio State University, Columbus, OH 43210, USA; Department of Animal Sciences, The Ohio State University, Wooster, OH 44691, USA; Department of Animal Sciences, The Ohio State University, Columbus, OH 43210, USA; Human Nutrition Program, The Ohio State University, Columbus, OH 43210, USA; Department of Animal Sciences, The Ohio State University, Columbus, OH 43210, USA

**Keywords:** carcass characteristics, cull cow, feeding, feedlot, ground corn, performance

## Abstract

Cull cows represent a significant percentage of revenue received from the U.S. beef industry; however, cull cows are heavily price discounted at time of slaughter. This experiment’s objective is to evaluate different feeding strategies and their effects on body condition score, subcutaneous fat color, and carcass yield and quality traits in cull cows. The central hypothesis is feeding a high-energy diet, with low levels of vitamin A, for 56 d will improve animal performance, carcass yield, and quality traits in addition to capturing the point (rate) of the conversion of yellow to white subcutaneous fat. In the present experiment 98 Angus crossbreed cows were utilized. Cows were fed either low vitamin A (**LVA**) diet consisting of whole shelled corn, soybean hulls, soybean meal, and a mineral-vitamin supplement or high vitamin A (**HVA**) diet, formulated using whole shelled corn, fescue hay, dry distiller grains with soluble, and a mineral-vitamin supplement for 56 d. During the 56 d feeding period, body weights and condition scores, and subcutaneous adipose samples were collected every 14 d. On day 56, cattle were slaughtered; 48 h postmortem carcass characteristics and objective color scores (subcutaneous adipose tissue) were recorded and a sample of the longissimus dorsi lumborum was collected. Subcutaneous adipose tissue samples were utilized to record subjective color scores and then ground to be analyzed for β-carotene concentration. The longissimus dorsi lumborum samples (2.54 cm slices) were removed for Warner-Bratzler shear force (**WBSF**) and pH testing. Data were analyzed using the MIXED procedure of SAS. Feeding cull cows LVA resulted in differences in subcutaneous carcass fat color (*P* = 0.01) as well as b* values (*P* < 0.01) on day 56 compared with HVA. Subjective fat color scores were not different (*P* > 0.10) on day 0 or 14 but were different (*P* ≤ 0.05) on days 28, 42, and 56. Additionally, 9-cis-β-carotene concentration on day 56 were different (*P* = 0.05) between treatments. A trend was noticed for all-*trans*-β-carotene concentration (*P* = 0.10) on day 56 as well. Cull cow body weights were greater (*P* ≤ 0.04) when fed the LVA diet starting on days 14, 28, and 42; and a trend was noticed on day 56 (*P* = 0.09). Overall, cows fed the LVA treatment for 56 d exhibited decreased adipose yellowness and β-carotene concentrations as well as increased live weights.

## Teaser

A beef cull cow diet consisting of low levels of vitamin A will result closer to white colors in subcutaneous fat leading to fewer discounts for American beef producers selling cull cows.

## Introduction

Cattle production is one of the most important industries in the United States. According to the National Agricultural Statistics Service (NASS), in 2015, cattle production was ranked first in cash receipts. Cash receipts are used as a means of showing “revenues from the sale of agricultural commodities, program payments from government agencies, and payments from private crop and livestock insurance programs” (USDA, 2016). Beef cattle production in the United States is a complex system involving many facets, beginning with cow calf producers, and eventually ending with slaughter.

Most of the beef in the United States originates from fed beef ranging in age from 14 to 18 mo. However, cull cattle contribute a significant share to the overall beef production numbers, up to 19% of cattle slaughtered in the United States (Woerner, 2010). A few reasons cows are sent to slaughter, include not being bred, old age, physical defects, and/or producing inferior calves relative to the herd index. Traditionally, most producers will sell their culled cows during the fall, as most cows calve in the spring, are bred during the summer, and are evaluated for being pregnant during the fall. However, this results in an over-supply of cull cows during a short period of time, resulting in prices being lowest during this time of the year, with the vast majority of cull cows sold through auction barns, producers become price takers, resulting in a decrease in potential farm income. With the sale of cull cows contributing 20% of farm revenue (Woerner, 2010), producers need to evaluate alternative ways to improve cull cow value while not losing money. A few simple tactics include having healthy cows based on body condition scores; feeding of high-energy diets; targeting a market cow class; and understanding the price index (an index of variations in prices of goods and services) (Boyles, 2015).

Cull cows are significantly price discounted at slaughter due to advanced physiological age and their high prevalence of exhibiting yellow fat color, due to high levels of vitamin A precursors present in forage (Kerth et al., 2007). Several studies have shown a correlation between a forage-based diet and advanced skeletal maturity on palatability, fat color, and percent sellable product. These factors combined could result in undervalued end products from cull cattle, with much of the carcass being ground. However, more cow slaughter facilities are selling whole muscle cuts to the food service industry resulting in increased revenues. Therefore, there is a need for information on how to improve palatability and whiteness of fat in the cull cow sector.

The central hypothesis of the current experiment is feeding a high-energy diet, with low levels of vitamin A, for 56 d will improve animal performance, carcass yield, and quality traits in addition to capturing the point (rate) of the conversion of yellow to white subcutaneous fat. This experiment’s objective is to evaluate feeding diets with different concentrations of vitamin A, and its precursor, on body condition score, subcutaneous fat color, carcass yield, and quality traits in cull cows. An understanding of the impact of different diets, which differ on levels of vitamin A, on performance, carcass traits and subcutaneous fat color may allow producers to more effectively feed cull cows to improve the market for meat from cull cows and maximize revenues.

## Materials and Methods

Cull-cows used for this study were selected by the managing team at two Ohio Agricultural Research and Development Center beef facilities. Cows were checked every October for pregnancy, those that were culled were utilized in the current study.

### Animals and Feed

All animal procedures were approved by the Ohio State University Institute of Animal Care and Use Committee (#2017A00000083) and followed the guidelines recommended in the Guide for the Care and Use of Agricultural Animals in Agricultural Research and Teaching (FASS, 2010).

Ninety-eight Angus crossbreed cull cows (ranging 3 to 6 yr) were used in two consecutive years with a feeding time of 56 d per year under a clean bunk management. Each year cows were fed in two different facilities at The Ohio State University, and each treatment was similarly represented per facility. Cows were sorted by body condition score and then divided into two feed treatments: low vitamin A (**LVA**) concentration and high vitamin A (**HVA**) concentration. The LVA diet was formulated using whole shelled corn (25%), soybean hulls (60%), soybean meal (10%), and a mineral–vitamin supplement (5%). The HVA diet was formulated using whole shelled corn (25%), fescue hay (45%), dried distillers’ grains with solubles (**DDGS**; 25%), and a mineral–vitamin supplement (5%). Ingredients chosen were determined by vitamin A levels (Pickworth et al., 2012). Soybean hulls were used as a forage source that contains vitamin A equivalents of 44 UI/kg of dry matter (**DM**), compared to fescue hay, the forage source of the HVA that contains 2,900 UI/kg of DM. As a protein source soybean meal has a vitamin A precursor concentration of 59 UI/Kg of DM, compared with 800 UI/kg of DM in the DDGS (Pickworth et al., 2012). The mineral–vitamin supplements were formulated without the addition of vitamin A for the LVA or with additional vitamin A for the HVA, supplying 41,771 IU/kg of supplement in the HVA.

For the first year, 59 cows were used; 44 cows on location 1 were assigned to two treatment pens consisting of 22 cows to HLA and 22 cows to LVA; and 15 cows on location 2 were assigned to two treatment pens consisting of 8 cows to HLA and 7 cows to LVA. For the second year, 39 cows were used; 22 cows on location 1 were assigned to two treatment pens consisting of 11 cows to HLA and 11 cows to LVA; and 17 cows on location 2 were assigned to two treatment pens consisting of 8 cows to HLA and 9 cows to LVA.

### Cow Evaluation Factors

On days 0, 14, 28, 42, and 56 cows were first weighed using a chute scale followed by the removal of fat biopsies conducted by a veterinarian. Body condition scores were recorded as cows walked to their pens. Body conditions scores ranged from a 1 to 9 scale, with 1 being extremely thin and 9 being obese (Rasby et al. 2014).

Fat biopsies were taken on days 0, 14, 28, 42, and 56. An area of 5 × 5 cm was designated close to the rump area by the base of the tail alternating sides of the animal on each biopsy day. The designated area was shaved, scrubbed followed by an application of 5 mL of lidocaine was used locally to reduce sensitivity. After three to four min., using a sterile scalpel, a 5-cm cut was made to remove a 2 × 2 × 2 cm subcutaneous adipose tissue sample for color evaluation. Upon removal of subcutaneous fat samples, samples were rinsed using sterile water, dried off, placed in a whirl-pak bag then stored in a Styrofoam cooler filled with dry ice. Samples were then transported in coolers back to the Department of Animal Sciences at The Ohio State University and placed in a −62 °C freezer.

### Beef Carcass Evaluation and Analysis

To assure subjective evaluations were consistent, three practice sessions were held in a large-scale meat plant harvesting a mixture of fed beef and cows. Visual evaluation of fat color was conducted by three animal science personnel who used a 1 to 5 number scale published by the American Meat Science Association (AMSA, 2012). Fat color scale ranged from 1 to 5 with color descriptors to include: 1, white; 2, creamy white; 3, slightly yellow; 4, moderately yellow; and 5, yellow.

Upon day 56, cows were sent to one of three locations for slaughter: a local federally inspected meat processor, the Ohio State University federally inspected meat laboratory, or a large scale federally inspected cow plant. Cows from location 1 were all harvested at a large-scale cow plant, whereas, cows from location 2 were split between a local meat processor and the OSU meat laboratory. This was based on size and space as well as processor’s availability to slaughter. The number of animals per treatment per year varied depending on the availability of the harvest plans to slaughter those animals at that time. Upon 48 h postmortem, carcasses were evaluated for: subjective subcutaneous fat color, yield (subcutaneous fat, ribeye area, kidney, pelvic and heart; and hot carcass weight), and quality grades (marbling score, skeletal maturity, and lean maturity) evaluated at the 12th/13th rib interface. Data was collected by two trained Ohio State personnel who utilized a United States Department of Agriculture (USDA) ruler, a beef ribeye dot grid, and USDA Marbling cards as points of reference. Objective color scores of subcutaneous fat were recorded for L* (lightness), a* (redness), and b* (yellowness) values. Objective color scores were recorded using a minolta colorimeter (Konica Minolta Colorimeter CR-410, 50 mm aperture, D65 illuminant; Minolta Company, Ramsey, NJ). Readings were taken in triplicate on the posterior-dorsal end of the shortloin. Objective color readings were only taken on day 56 due to too small fat biopsy sample sizes taken. Muscle samples (10.16 cm thick) were removed from the left sides of each carcass originating from the anterior portion of the shortloin area starting at the 12th/13th rib interface. Muscle samples were vacuum packaged and transported under refrigerated conditions to the Animal Sciences building at The Ohio State University where they then were sliced into 2.54 cm slices, upon arrival, re-packaged then stored at −80 °C for further analysis.

### Muscle Sample Analysis

Prior to analysis, muscle sample slices were first thawed followed by removing the *longissimus lumborum* (loin eye) was separated for future analysis (muscle pH and tenderness).

For muscle pH, meat samples were first frozen in liquid nitrogen and then ground into a fine powder before two aliquots of 100 mg apiece were placed in 1.5 mL plastic tubes. Muscle pH was performed according to Bendall (1973). The two 100 mg aliquots of powdered muscle sample were homogenized in an ice-cold measurement buffer (5 mM iodoacetic acid and 150 mM KCl (pH 7.0)) at a 1:8 ratio. The muscle and buffer mixtures were warmed to 25 °C, centrifuged, and measured with a pH probe (Bendall, 1973). Samples were measured in duplicate.

Warner-Bratzler shear force (WBSF) followed the Caine et al. (2003) procedure, where a 2.54-cm muscle sample was thawed to 4 °C overnight prior to cooking. On the day of cooking, muscle samples were cooked to an internal temperature of 71 °C using a clamshell grill as the cooking instrument. Temperature was measured using a thermocouple placed in the geometric center of each sample. Upon cooking, samples were cooled to an internal temperature of 4 °C overnight. Post 24 h, six cores (19 mm in diameter) were taken parallel to muscle fibers then sheared using a TAXT-plus machine (TA.XT plus Connect, Texture Technologies, Hamilton, MA). A Warner-Bratzler shear blade was used to determine peak force (N) and toughness (kg) (Caine et al. 2003).

### Carotenoid Analysis

Carotenoid analysis was conducted by powdering frozen adipose (200 mg) in liquid nitrogen before mixing with 0.5 mL methanol, 5 mL hexane, and 5 mL of a 0.1% ethanolic sodium hydroxide solution. Samples were continuously stirred at 4 °C 1 h, then 5 mL of distilled water was added and the vial vortexed to induce phase separation. The resulting supernatant was pipetted off and dried under nitrogen gas. Samples were stored dry at -80°C prior to high performance liquid chromatography-photodiode array (HPLC-PDA) analysis.

Samples were analyzed using an HPLC (Waters 2996, Waters Corporation, Milford, MA) equipped with a Waters 2996 PDA, using a C-30 column (150 × 4.6mm, 3um particle size, YMC America, Allentown, PA). Samples were redissolved with 100 µL of MTBE and 100 µL of methanol, and vortexed for five seconds before being filtered through a nylon syringe filter (0.22 µm pore size). A gradient elution method was employed using solvent A (MeOH:H20, 80:20, v/v) with 0.1% aq. formic acid and Solvent B (MTBE: MeOH: H20, 78:20:2, v/v) with 0.1% aq. formic acid added. The gradient was as follows: beginning at 5% B, increasing to 95% B over 10 min, holding at 95% B for 6 min, and returning and holding at 5% B over 4 min. The flow rate was 1.30 mL/min, column temperature 40°C and injection volume was 20 µL. Quantification was performed using an external calibration curve, generated with authentic β-carotene standard, and comparing relative peak areas of the curve to those in the samples to determine µg β-carotene/100 g of adipose tissue.

### Statistical Analysis

Data were analyzed as a complete block design, using the PROC MIXED of SAS (SAS Inst. Inc. Cary, NC). The model included dietary treatment, time, and time by treatment interaction as the fixed variables, whereas block (year and facility) and pen within each block was a random variable. Means separation for interaction of treatment and time were conducted using the SLICE option of SAS. Due to the initial effect of age and BCS, initial outcomes were used as co-variables. Significant differences were declared at *P* ≤ 0.05, whereas tendencies were declared at *P* ≤ 0.10.

## Results and Discussion

### Weight and Body Condition Score

**Figure 1.**
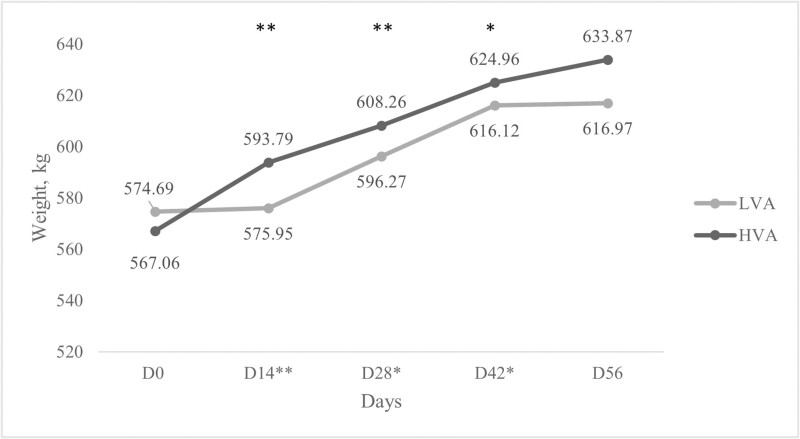
Least square means on the effect of days on feed on weight (kg) of cull cows fed diets formulated with low vitamin A (LVA) or high vitamin A (HVA) for 56 d. Days with * indicate significance (*P* ≤ 0.05), days with ** indicate *P* ≤ 0.01. Standard error of the mean was 42.8.

**Figure 2.**
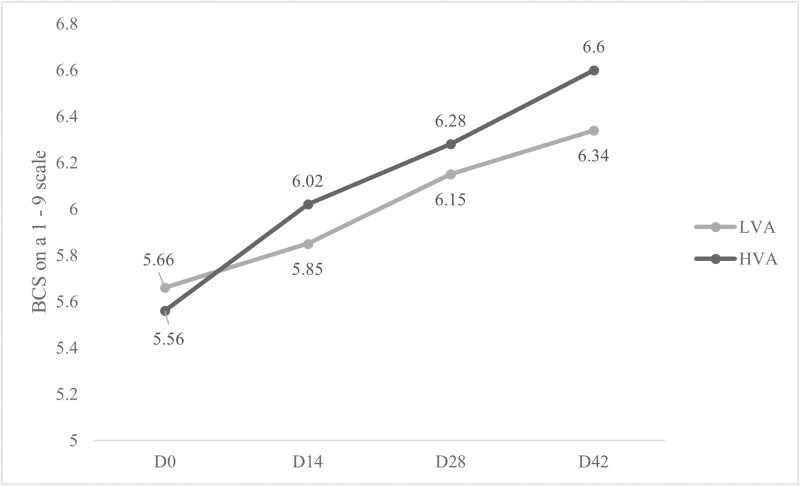
Least square means on the effect of days on feed on body condition score of cull cows fed diets formulated with low vitamin A (LVA) or high vitamin A (HVA) for 56 d. Days with * indicate significance at *P* ≤ 0.05, days with ** indicate *P* ≤ 0.01. Standard error of the mean was 0.34.

There was a treatment by time interaction (*P* < 0.01; Fig. 1) for body weights, where weights of cull cows were significantly affected on days 14 (*P* < 0.01), 28 (*P *= 0.01), and 42 (*P* = 0.04) and a trend was noticed on day 56 (*P* = 0.09) with cows in the HVA treatment weighing more. Body condition scores (BCS; Fig. 2), however, were not affected (*P* > 0.1) on day 14, 28, or 42; BCS was not assessed on day 56 prior to slaughter. These findings conflict with results found in previous studies (Gorocica-Buenfil et al. 2008; Pickworth et al. 2011, 2012) which noted no significant differences in growing weight of Angus-based steers fed treatments consisting of low and HVA diets. This could be a result of differences in diet formulations, in each of the above studies base diets for all treatments were the same, with the difference being supplemental vitamin A. Conversely, in this trial, difference in provitamin A concentration was due to completely different diet formulations between treatments. Additionally, age of animals, sex of animals, and time on feed between the above-mentioned studies and the current study were notably different.

### Carcass Characteristics

Carcass characteristics were not affected (*P* ≥ 0.32) by treatment (Table 1). However, subcutaneous fat color exhibited a treatment difference (*P* = 0.01; Table 1), with the LVA treatment presenting less overall yellowness (2.22 vs. 2.58). While these values are different, in practice the difference in color scores of 2.22 and 2.58 would scarcely be noticeable to the human eye. Carcasses of main concern usually fall within the 3 (slightly yellow) -5 (yellow) color score range. Determination of color scores and subsequent application of premiums and discounts to carcasses is up to the discretion of graders at individual plants that can vary widely between plants (Hunt and King, 2012). This result means a potential increase in feed time may be needed to note visual differences between treatments. This finding supports previous studies (Gorocica-Buenfil et al., 2008; Pickworth et al., 2011) reporting subcutaneous fat, ribeye area, hot carcass weight, marbling score, and quality grade were not affected by diets supplemented with LVA or HVA when fed to Angus-based steers. Pickworth et al. (2012), however, found that vitamin A supplementation fed to Angus-crossbreed steers for 184 d significantly affected hot carcass weights, marbling scores, and quality grades. Gorocica-Buenfil et al. (2007a) noted no significant differences between subcutaneous fat, ribeye area, or hot carcass weight, but did find differences in marbling score and quality grade between Angus-cross steers fed high and LVA concentrations. Additionally, Gorocica-Buenfil et al. (2007b) noted no significant differences in carcass traits between Holstein steers on a vitamin A restricted diet for 112 or 243 d. These conflicting finds could be attributed to differences in genetics, sex, and age of animals used in each study.

**Table 1.** Least square means of carcass data and quality traits of cow carcasses fed diets formulated with low vitamin A (LVA) or high vitamin A (HVA) for 56 d

	Treatment			
Items	LVA	HVA	SEM	*P-*value^1^
Cull cows	49	49		
PYG^2^	2.97	2.89	0.09	0.34
Subcutaneous fat, cm	1.02	0.93	0.09	0.32
Ribeye area, cm^2^	80.67	78.84	1.82	0.32
Hot carcass weight, kg	337.65	329.08	8.38	0.31
Skeletal maturity^3^	434.38	423.54	29.23	0.71
Lean maturity^4^	182.62	179.06	4.44	0.42
Overall maturity^5^	367.01	355.25	22.72	0.61
Marbling score^6^	410.25	404.69	16.34	0.73
Quality grade^7^	2.04	2.06	0.27	0.94
Subcutaneous fat color^8^	2.22	2.58	0.14	0.01

^1^ *P*-value: significance determined at ≤0.05, tendencies determined at ≤0.10.

^2^PYG, preliminary yield grade.

^3^Skeletal maturity: A (30 mo): 100 to 199, B (31 to 41 mo): 200 to 299, C (42 to 71 mo): 300 to 399, D (72 to 95 mo): 400 to 499, E (> 96mo) 500 to 599.

^4^Lean maturity: A: 100 to 199, B: 200 to 299, C:300 to 399, D: 400 to 499, E: 500 to 599.

^5^Overall maturity: A (30 mo): 100 to 199, B (31 to 41 mo): 200 to 299, C (42 to 71 mo): 300 to 399, D (72 to 95 mo): 400 to 499, E (> 96 mo) 500 to 599.

^6^Marbling score: practically devoid: 100 to 199, traces: 200 to 299, slight: 300 to 399, small: 400 to 499, modest/ moderate: 500 to 599, slightly abundant: 600 to 699, moderately abundant: 700 to 799, abundant: 800 - 899.

^7^Quality grade: cutter: 0.00 to 0.99, utility: 1.00 to 1.99, commercial: 2.00 to 2.99, standard: 3.00 to 3.99, select: 4.00 to 4.99, low choice: 5.00 to 5.99, top choice: 6.00 to 6.99, prime: 7.00 to 7.998.

^8^Subcutaneous fat color: 1, white; 2, creamy white; 3, slightly yellow; 4, moderately yellow; and 5, yellow.

### Subjective Fat Biopsy Color Score

**Figure 3.**
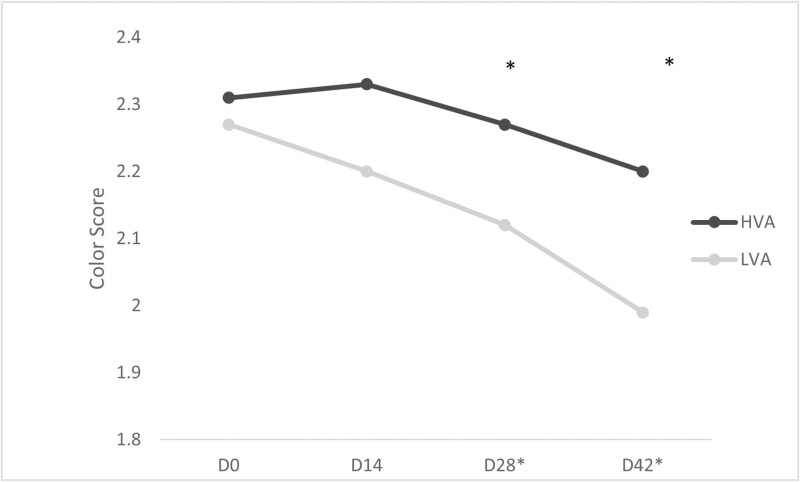
Least square means of subjective fat color scores of cull cows fed diets formulated with low vitamin A (LVA) or high vitamin A (HVA) for 56 d. Days with * indicate significance at *P *≤ 0.05, days with ** indicate *P* ≤ 0.01. Standard error of the mean was 0.146.

Subjective fat color scores are presented in Fig. 3. Subjective color scores were based on the American Meat Science Associations 1-5 fat color scoring scale. Fat color scores did not differ significantly between treatments on day 0 (*P* = 0.58) or day 14 (*P* = 0.13); however, a time by treatment difference was observed on day 28 (*P* = 0.05), 42 (*P* = 0.02), and 56 (*P* = 0.01). On day 56 subjective color scores of fat samples increased for both the HVA and LVA treatments compared to day 42. Cows on the HVA treatment had a score of 2.42, while the LVA treatment had a score of 2.12. The increase seen in both treatments color scores seen between days 42 and 56 could be result of difference in sample collection seen between slaughter locations. For instance, as part of a food safety intervention step, large scale plants commonly use a steam cabinet (ranging between 73 and 82 °C) to eliminate potential bacteria/pathogens right before sending beef carcasses into the chiller. It is concluded that the heat of the steam can, and will, affect fat color resulting in lighter shades. This was not discovered until after all data had been collected and analyzed. As a result, color scores for day 56 are not provided in Fig. 3.

### Objective Fat Color Score

There were no observed differences due to treatment for L* and a* (*P* ≥ 0.59; Table 2). The L*, representing lightness color value ranging from 0 to 100 as well as the a* representing red and green color values were not significant. The B*, which represents blue and yellow color values was different between the treatment groups (*P* < 0.01; Table 2). This corresponds with the subjective fat biopsy visual color scores presented previously (Fig. 3), which showed differences between treatments at days 28, 42, and 56. A study conducted by Rohrle et al. (2011) noted that crossbreed heifers fed a purely concentrate diet (low in provitamin A) were significantly less yellow (*P* < 0.05; Table 2) compared to crossbreed heifers fed a pasture, silage and pasture, or silage, pasture, and concentrate mix diet. Cattle in that study also had lighter subcutaneous adipose tissue when fed strictly concentrate (Rohrle et al., 2011). The disagreement between these two studies in terms of lightness could potentially be correlated to factors such as age, genetics, and diet formulation.

**Table 2.** Least square means of objective subcutaneous fat color scores of carcasses from cull cows fed diets formulated with low vitamin A (LVA) or high vitamin A (HVA) for 56 d

	Treatment			
Items	LVA	HVA	SEM	*P*-value^1^
Cull cows	49	49		
L*^2^	73.50	73.24	0.78	0.74
a*^3^	10.30	10.08	0.39	0.59
b*^4^	17.23	19.96	0.69	<0.01

^1^ *P*-value: significance determined at ≤0.05, tendencies determined at ≤0.10.

^2^L*: lightness.

^3^a*: redness.

^4^b*: yellowness.

### WBSF, peak force, and pH

Table 3 shows least square means for pH, WBSF and peak force of longissimus dorsi lumborum samples. No differences were noted between the LVA and HVA diets for WBSF (*P* = 0.92), peak force (*P* = 0.70), or pH (*P* = 0.32).

**Table 3.** Least square means of Warner-Bratzler shear force, peak force, and muscle pH of longissimus lumborum muscle samples from carcasses from cow fed diets formulated with low vitamin A (LVA) or high vitamin A (HVA) for 56 d

	Treatment			
Items	LVA	HVA	SEM	*P*-value^1^
Cull cows	49	49		
WBSF^2^, kg	4.78	4.81	0.26	0.92
Peak force^3^, kg*sec	11.95	12.18	0.60	0.70
pH	5.60	5.55	0.06	0.32

^1^ *P*-value: significance determined at ≤0.05, tendencies determined at ≤0.10.

^2^WBSF, Warner-Bratzler shear force, kg.

^3^Peak force: area under the curve, kg*sec.

### Carotenoid Concentration

Table 4 shows the least square means of both all-*trans*- and 9-*cis*-β-carotene in subcutaneous adipose samples collected on days 0 and 56. No differences were noted between the LVA or HVA treatments on day 0 for either all-*trans*-β-carotene (*P* = 0.37) or 9-*cis*-β-carotene (*P* = 0.42). On day 56, a difference was seen between LVA and HVA for 9-*cis*-β-carotene (*P *= 0.05), and a trend was noticed for all-trans-β-carotene (*P* = 0.10). These results support a previous study (Rohrle et al., 2011), which found significantly less β-carotene in subcutaneous fat samples of Charolais × Limousin cross heifers when feeding barley-based concentrate (0.09 µg/g) versus treatments consisting of pasture (0.54 µg/g), silage and pasture (0.49 µg/g), and silage, pasture, and concentrate (0.49 µg/g) for 11 mo. While Rohrle et al. (2011) showed significantly less β-carotene when heifers were being fed concentrate versus a silage or pasture diet and this study only identified a trend in all-*trans*-β-carotene and significance in 9-*cis*-β-carotene, this could be due to the duration of the study; 11 mo. versus 56 d.

**Table 4.** Least Square Means of β—Carotene concentration in subcutaneous fat from carcasses from cows fed diets formulated with low vitamin A (LVA) or high vitamin A (HVA) for 56 d.

	Treatment			
Items	LVA	HVA	SEM	*P*-value^1^
Cull cows	49	49		
D0 all-*trans*-β-carotene, µg/g	0.355	0.400	0.05	0.37
D56 all-*trans*-β-carotene, µg/g	0.200	0.242	0.03	0.10
D0 9-*cis*-β-carotene, µg/g	0.040	0.043	0.01	0.42
D56 9-*cis*-β-carotene, µg/g	0.013	0.020	<0.01	0.05

^1^ *P*-value: significance determined at ≤0.05, tendencies determined at ≤0.10.

In conclusion, overall, the study did support the feeding of an LVA diet for 56 d, from a statistical standpoint. However, when looking at the results of the LVA and HVA treatments, from an industry standpoint the differences seen in this study may not be adequate to support feeding cull cows a specialized LVA diet. Future research should be done to investigate the effects of other LVA diets and prolonged feeding time on subcutaneous adipose subjective and objective color scores as well as β-carotene in subcutaneous adipose tissue. Additional research should also be conducted to investigate the effect of time of year as well as breed type of cows.
